# Vestigial‐like family member 3 stimulates cell motility by inducing high‐mobility group AT‐hook 2 expression in cancer cells

**DOI:** 10.1111/jcmm.17279

**Published:** 2022-04-02

**Authors:** Naoto Hori, Yuki Takakura, Ayumi Sugino, Shuto Iwasawa, Kota Nomizo, Naoto Yamaguchi, Hiroyuki Takano, Noritaka Yamaguchi

**Affiliations:** ^1^ Laboratory of Molecular Cell Biology Graduate School of Pharmaceutical Sciences Chiba University Chiba Japan; ^2^ Department of Molecular Cardiovascular Pharmacology Graduate School of Pharmaceutical Sciences Chiba University Chiba Japan

**Keywords:** EMT, signal transduction, TEAD, TGF‐β, VGLL3

## Abstract

Vestigial‐like family member 3 (VGLL3) is a cofactor for TEA domain transcription factors (TEADs). Although VGLL3 is known to be highly expressed and stimulate cell proliferation in mesenchymal cancer cells, its involvement in mesenchymal phenotypes is largely unknown. In this study, we found that VGLL3 promotes epithelial‐to‐mesenchymal transition (EMT)‐like phenotypic changes. We found that A549 human lung cancer cells stably expressing VGLL3 exhibit spindle‐like morphological changes, reduction in the epithelial marker E‐cadherin and induction of the mesenchymal marker Snail. Notably, VGLL3‐expressing cells exhibited enhanced motility. The DNA‐binding protein high‐mobility group AT‐hook 2 (HMGA2) was found to be a target of the VGLL3‐TEAD4 complex, and HMGA2 knockdown repressed EMT‐like phenotypic changes in VGLL3‐expressing cells. VGLL3‐dependent phenotypic changes are involved in transforming growth factor‐β (TGF‐β)‐induced EMT progression. VGLL3 or HMGA2 knockdown repressed the motility of the mesenchymal breast cancer MDA‐MB‐231 cells. Importantly, high levels of VGLL3 expression were shown to have a positive correlation with poor prognosis in various human cancers, such as breast, colon, ovarian, head and neck, pancreatic, renal, gastric and cervical cancers. These results suggest that VGLL3 promotes EMT‐like cell motility by inducing HMGA2 expression and accelerates cancer progression.

## 1 INTRODUCTION

Epithelial‐to‐mesenchymal transition (EMT) is a process that converts adherent epithelial cells into motile mesenchymal cells during embryonic development and tumour progression. E‐cadherin, encoded by *CDH1*, is a crucial protein that mediates cell–cell adhesion in epithelial cells.^1^, ^2^, ^3^ A reduction in E‐cadherin expression is an important step in the progression of EMT, and E‐cadherin expression is suppressed by transcriptional repressors of *CDH1*. The major transcriptional repressors of *CDH1* are the zinc finger factors Snail and Slug. These two proteins bind to the E‐box sequences present in the *CDH1* enhancer region and strongly repress *CDH1* expression.^4^, ^5^

Transforming growth factor‐β (TGF‐β) is a multifunctional cytokine that regulates various cellular events, including differentiation, proliferation, apoptosis and EMT.^6^, ^7^, ^8^ TGF‐β transduces cellular signals by bridging two pairs of receptors (type I and type II receptors) that have serine/threonine kinase activities. TGF‐β binding primarily activates type II receptor‐mediated phosphorylation of type I receptors. The type I receptors mainly phosphorylate Smad2 and Smad3, the central transcription factors involved in TGF‐β signalling. Phosphorylated Smad2 and Smad3 bind to the homologous protein Smad4 and translocate into the nucleus, resulting in the expression of a variety of target genes.^6^, ^7^, ^8^ Although Snail and Slug are widely accepted as downstream targets of TGF‐β Smad signalling involved in EMT progression,^9^ there are cases where the expression of each gene is not sufficient to induce strong EMT.^10^ Therefore, it is likely that there are TGF‐β signalling regulators involved in EMT progression that are still unidentified.

The vestigial‐like family (VGLL) members are orthologs of the vestigial gene product in *Drosophila* and are composed of four homologs. VGLL members are thought to function as transcriptional cofactors by binding to the TEA domain transcription factors (TEADs) via the highly conserved Tondu (Tdu) domain.^11^, ^12^ Previous studies have shown that expression of VGLL3, a member of the VGLL family, is induced by TGF‐β stimulation.^13^ Furthermore, *VGLL3* gene amplification has been observed in several types of sarcomas, and VGLL3 has been shown to promote tumour cell growth.^14^, ^15^, ^16^ Therefore, it is reasonable to speculate that VGLL3 is involved in EMT phenotypes during cancer progression. The present study was designed to analyse the relationship between VGLL3 and EMT phenotypes in cancer cells.

## 2 EXPERIMENTAL PROCEDURES

### 2.1 Plasmids

The cDNA encoding human *VGLL3* (a myc tag was added to the N‐terminus) or *TEAD4* was subcloned into the pIRESpuro3‐CAG vector as described previously.^15^ The *HMGA2* reporter plasmid was constructed as follows. The EGFP expression vector (pcDNA/EGFP)^17^ was digested with NruI and KpnI, and the cytomegalovirus (CMV) promoter was removed. The cDNA fragments corresponding to the *HMGA2* promoter region (between −3024 and +77 from the transcription start site of the *HMGA2* gene predicted from RefSeq NM_003483.6) were generated by PCR using genomic DNA isolated from A549 cells. The fragments were then digested with EcoRV and KpnI and subcloned into the pcDNA/EGFP vector. The following primers were used for PCR: 5′‐AAAGATATCGCTGAGTAAAGAGGGGAGCCCATT‐3′ (sense) and 5′‐TTTGGTACCCGGAGAGTCGGAGGGGGACGGGCTG‐3′ (antisense). The pCAG/TetR vector, which constitutively expresses tetracycline repressor (TetR), was used to assess transfection efficiency.^15^

### 2.2 Western blotting and antibodies

Western blotting was performed using an enhanced chemiluminescence kit (Merck Millipore), as described previously.^15^ The following antibodies were used: myc (PL14) and β‐actin (M177‐3) from MBL (Nagoya, Japan); E‐cadherin (#3195), N‐cadherin (#13116), Smad2/3 (#8685), phospho‐Smad2 (#3108), phospho‐Smad3 (#9520), Snail (#3879), Slug (#9585), HMGA2 (#5269) from Cell Signaling Technology (Beverly, MA); TEAD4 (ab58310) from Abcam (Cambridge, UK); GFP (mFX75) from Fujifilm Wako (Tokyo, Japan); TetR (TET01) from MoBiTech (Cambridge, MA, USA); and TWIST (25465–1‐AP) from Proteintech (Rosemount, IL, USA). Horseradish peroxidase (HRP)‐F(ab’)2 secondary antibodies were purchased from GE Healthcare (Waukesha, WI, USA). Protein bands were analysed using a ChemiDoc XRS+image analyzer (Bio‐Rad, Hercules, CA, USA). The intensity of bands was measured by Quantity One software (Bio‐Rad). Quantitative ratios of E‐cadherin, N‐cadherin, Snail, Slug, HMGA2 or TEAD4 to actin and EGFP to TetR, which were calculated based on the data, are shown as relative values.

### 2.3 Cells and transfection

A549 and MDA‐MB‐231 cells were cultured in Dulbecco's modified Eagle's medium (DMEM) supplemented with 5% foetal bovine serum at 37°C. A549 cells stably expressing myc‐VGLL3 or an empty vector were established as previously described.^15^ To transfect plasmids, cells were seeded in a 35‐mm (60‐mm) culture dish and transiently transfected with 1 μg (3 μg) of plasmid DNA using Lipofectamine 2000 (Thermo Fisher Scientific, Fair Lawn, NJ), according to the manufacturer's instructions. The siRNAs (5 nM) were transfected using the reverse transfection method with Lipofectamine RNAiMax (Thermo Fisher Scientific, Somerset, NJ, USA) according to the manufacturer's instructions. Silencer select siRNAs specific for *VGLL3* #1 (s52380), *VGLL3* #2 (s52378), *SNAI1* (s13186), *TEAD4* (s13964), *HMGA2* #1 (s15616), *HMGA2* #2 (s194864) and mock siRNA (4390843) were purchased from Thermo Fisher Scientific. Human recombinant TGF‐β1 was purchased from PeproTech (Rocky Hill, NJ, USA).

### 2.4 Quantitative real‐time PCR (qPCR) analysis

The qPCR was performed as described previously.^15^ In brief, total RNA was isolated from cells using RNAiso plus reagent (TaKaRa, Shiga, Japan), and cDNA was synthesized from 0.5 μg of each RNA preparation using the ReverTra Ace qPCR RT Kit (TOYOBO, Tokyo, Japan), according to the manufacturer's instructions. The primers used for PCR were as follows: *GAPDH*, 5′‐ACCACAGTCCATGCCATCAC‐3′ (sense) and 5′‐TCCACCACCCTGTTGCTGTA‐3′ (antisense); *CDH1*, 5′‐CGAGAGCTACACGTTCACGG‐3′ (sense) and 5′‐GGGTGTCGAGGGAAAAATAGG‐3′ (antisense); *CDH2*, 5′‐AGCCAACCTTAACTGAGGAGT‐3′ (sense) and 5′‐GGCAAGTTGATTGGAGGGATG‐3′ (antisense); *SNAI1*, 5′‐TCGGAAGCCTAACTACAGCGA‐3′ (sense) and 5′‐AGATGAGCATTGGCAGCGAG‐3′ (antisense); *SNAI2*, 5′‐TGTGACAAGGAATATGTGAGCC‐3′ (sense) and 5′‐TGAGCCCTCAGATTTGACCTG‐3′ (antisense); *HMGA2*, 5′‐ACCCAGGGGAAGACCCAAA‐3′ (sense) and 5′‐CCTCTTGGCCGTTTTTCTCCA‐3′ (antisense); and *TWIST1*, 5′‐GTCCGCAGTCTTACGAGGAG‐3′ (sense) and 5′‐GCTTGAGGGTCTGAATCTTGCT‐3′ (antisense). After initial denaturation at 95°C for 1 min, PCR was performed for 40 cycles (15 s at 95°C and 45 s at 60°C) using the Thunderbird SYBR Green Polymerase Kit (TOYOBO) and the Eco Real‐Time PCR System (Illumina, San Diego, CA).

### 2.5 Wound healing assay

Cells were seeded into 6‐well plates at a density of 500,000 cells/well. They were then transfected with siRNAs (5 nM) using the reverse transfection method with Lipofectamine RNAiMax (Thermo Fisher Scientific, Somerset, NJ, USA) according to the manufacturer's instructions. At 48 h after transfection, cells were transfected with additional siRNAs using the forward transfection method with Lipofectamine RNAiMax. When the cell confluence reached approximately 100% at approximately 48‐h post‐second transfection, scratch wounds were made by scraping the cell layer across each culture plate with a 200‐μl pipette tip. After wounding, the debris was removed by washing the cells with fresh medium. The wound areas were measured at 0, 8 or 12 h after wounding with ImageJ, and microscopic images of the cells were captured. Migrated areas were calculated as follows: migrated area (%) = (wound area at 0 h [pixel] – wound area at 8 or 12 h [pixel]) × 100 [%]/wound area at 0 h [pixel]. The experiments were performed in triplicate.

### 2.6 Chromatin immunoprecipitation assay

The ChIP assay was performed as described previously.^18^, ^19^ Chromatin solution (2 ml) was incubated overnight with 2 μg of anti‐myc antibodies (PL14) and control mouse antibody (MOPC21; SIGMA‐Aldrich, St Louis, MO). DNA was purified and subjected to qPCR analysis as described above. We used the following PCR primers: amplicon 1 (between −2023 and −1821 from the transcription start site of the HMGA2 gene predicted from RefSeq NM_003483.6), 5′‐AGCAGCCTGAAAACAAGTGG‐3′ (sense) and 5′‐GGGGAGTCACTGAGGAGTTC‐3′ (antisense); amplicon 2 (between −660 and −484), 5′‐GCATGTCTCCGTGTATGTGC‐3′ (sense) and 5′‐GAGCCAACACTTTGCAGGAA‐3′ (antisense). Per cent input values were calculated by comparing *Ct* values of input and immunoprecipitated fractions and were shown as ratios relative to those of control samples.

## 3 RESULTS

### 3.1 VGLL3 promotes EMT‐like phenotypic changes

**FIGURE 1:**
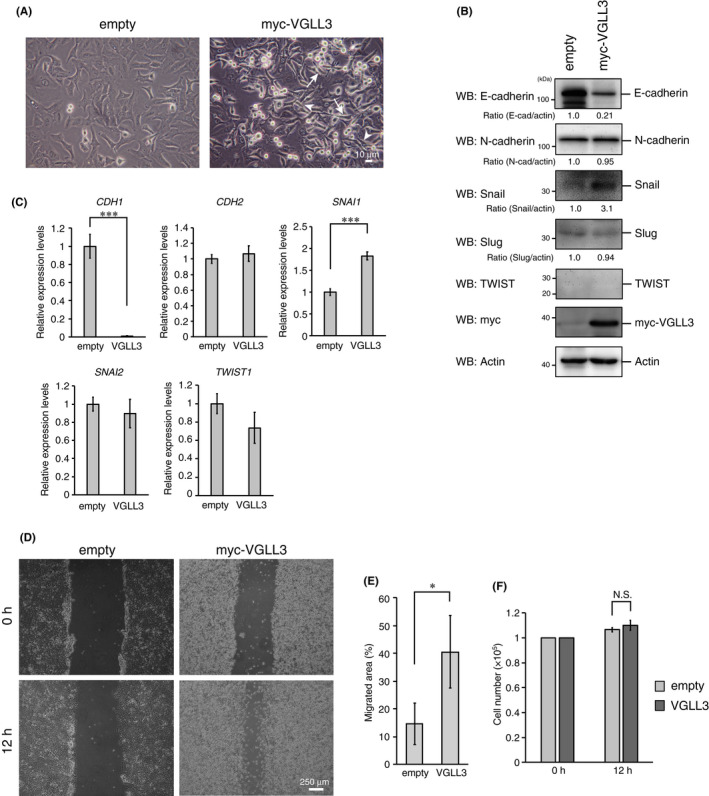
VGLL3 expression induces epithelial‐to‐mesenchymal transition (EMT)‐like phenotypic changes. (A) A549 cells stably expressing the empty vector or myc‐VGLL3 are visualized by phase‐contrast microscopy. Arrows and arrow heads show spindle‐like cells and piling‐up cells, respectively. (B) A549 cells stably expressing the empty vector or myc‐VGLL3 were analysed using Western blotting with the indicated antibodies. (C) A549 cells stably expressing the empty vector or myc‐VGLL3 were analysed for *CDH1* (E‐cadherin), *CDH2* (N‐cadherin), *SNAI1* (Snail), *SNAI2* (Slug) and *TWIST1* (TWIST) mRNA expression using qPCR. *GAPDH* mRNA expression was used for data normalization. The expression level of each mRNA in the control cells was set to 1. Results are expressed in terms of mean ± SD (*n* = 3). Asterisks indicate the statistical significance (***p < 0.001) calculated using Student's *t*‐test. (D) Representative images of wounded areas of A549 cells stably expressing the empty vector or myc‐VGLL3 at 0 h and 12 h after wounding. (E) Migrated areas at 12 h after wounding. Error bars show SD (*n* = 3). Asterisk indicates the statistical significance (*p < 0.05) calculated using Student's *t*‐test. (F) A549 cells stably expressing the empty vector or myc‐VGLL3 were seeded at 1 × 10^5^ cells/well, respectively, and cultured for the indicated times. After the incubation, cell numbers were counted, and viable cells numbers were plotted. N.S., not significant

We have previously established a cell line stably expressing myc‐VGLL3 using human lung cancer A549 cells.^15^, ^16^ These cells show spindle‐like morphological changes (Figure 1A, arrows) and piling‐up characteristics (Figure 1A, arrow heads). Given that these phenotypic changes are similar to those in EMT cells, VGLL3‐stable cells were expected to exhibit EMT‐like characteristics. Notably, E‐cadherin expression was strongly downregulated, whereas Snail expression was significantly upregulated at both the mRNA and protein levels in these cells (Figure 1B,C). The expression of other mesenchymal markers, N‐cadherin and Slug, was not affected (Figure 1B,C). While TWIST is another crucial EMT inducer in cancer cells,^20^ its induction was not detected at the mRNA or protein level (Figure 1B,C). Wound healing assays showed that VGLL3‐expressing cells had an enhanced healing rate of the cell layer wounds (Figure 1D,E). Cell growth rates of control and VGLL3‐expressing cells were not different under the experimental conditions (12‐h incubation) (Figure 1F), suggesting that the enhanced healing rate indicates enhanced cell motility. Thus, these results suggest that VGLL3 expression promotes EMT‐like phenotypic changes.

### 3.2 HMGA2 mediates VGLL3‐induced EMT‐like phenotypic change

**FIGURE 2:**
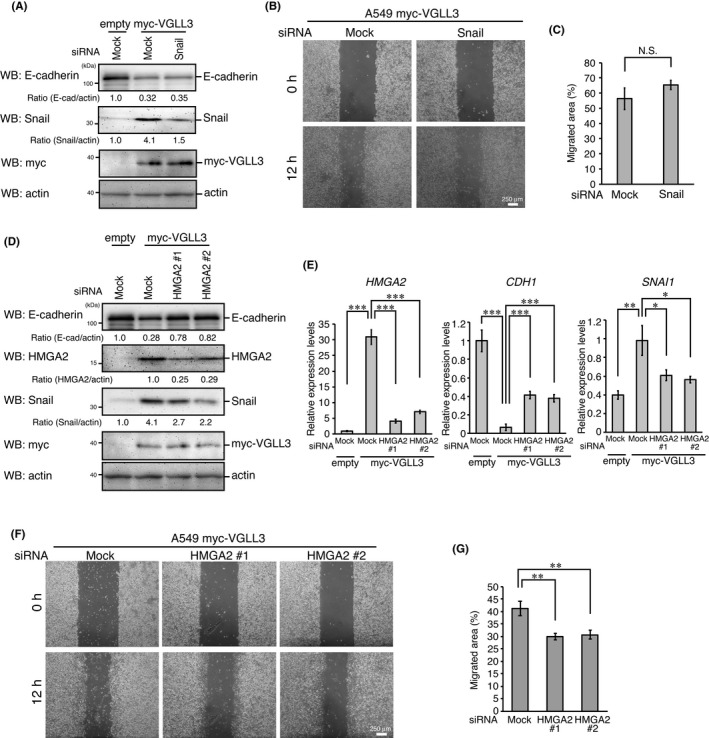
VGLL3‐induced EMT‐like phenotypic changes depend on HMGA2. (A, D) A549 cells stably expressing the empty vector or myc‐VGLL3 were transfected with the indicated siRNAs for 48 h. After incubation, cells were analysed by Western blotting with the indicated antibodies. (B, F) Representative images of wounded areas of A549 cells stably expressing myc‐VGLL3 transfected with the indicated siRNAs at 0 h and 12 h after wounding. (C, G) Migrated areas at 12 h after wounding. Error bars show SD (*n* = 3). N.S., not significant. (E) A549 cells stably expressing the empty vector or myc‐VGLL3 were analysed for *HMGA2*, *CDH1* (E‐cadherin) and *SNAI1* (Snail) mRNA expression using qPCR, as in Figure 1C. Asterisks indicate the statistical significance (***p < 0.001; **p < 0.01; *p < 0.05) calculated using Student's *t*‐test

Because VGLL3‐expressing cells showed enhanced expression of the EMT‐inducer Snail, we examined the involvement of this protein in VGLL3‐induced phenotypic changes. Although siRNA transfection reduced Snail expression, no apparent change was observed in E‐cadherin expression or cell motility (Figure 2A–C), suggesting that Snail is not the main inducer of EMT‐like phenotypic changes in VGLL3‐expressing cells.

Previous studies have shown that VGLL3 regulates mesenchymal differentiation.^21^, ^22^ The DNA‐binding protein high‐mobility group AT‐hook 2 (HMGA2) is also known to regulate mesenchymal differentiation and EMT.^23^, ^24^, ^25^ Thus, we examined the involvement of HMGA2 in EMT‐like phenotypes in VGLL3‐expressing cells. VGLL3‐expressing cells showed elevated expression of HMGA2 (Figure 2D,E). Notably, HMGA2 knockdown significantly increased E‐cadherin expression and suppressed cell motility in VGLL3‐expressing cells (Figure 2D–G). Snail expression was also suppressed by HMGA2 knockdown (Figure 2D,E). These results suggest that HMGA2 mediates VGLL3‐induced EMT‐like phenotypes.

### 3.3 VGLL3 regulates HMGA2 expression via TEAD4

**FIGURE 3:**
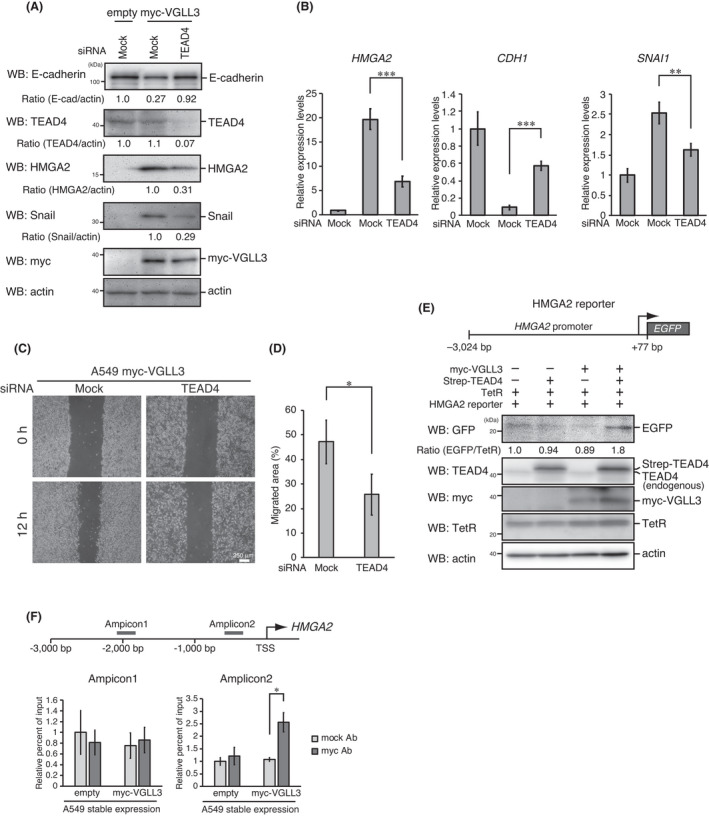
HMGA2 is a target of VGLL3‐TEAD4 complex. (A) A549 cells stably expressing the empty vector or myc‐VGLL3 were transfected with the indicated siRNAs for 48 h. After incubation, cells were analysed by Western blotting with the indicated antibodies. (B) A549 cells stably expressing the empty vector or myc‐VGLL3 were analysed for *HMGA2*, *CDH1* (E‐cadherin) and *SNAI1* (Snail) mRNA expression using qPCR, as in Figure 2(E). (C) Representative images of wounded areas of A549 cells stably expressing myc‐VGLL3 transfected with the indicated siRNAs at 0 h and 12 h after wounding. (D) Migrated areas at 12 h after wounding. Error bars show SD (*n* = 3). Asterisk indicates the statistical significance (*p < 0.05) calculated using Student's *t*‐test. (E) A549 cells were co‐transfected with (+) or without (–) the indicated plasmids for 24 h. After the incubation, cells were analysed by Western blotting with the indicated antibodies (lower). The schematic diagram of the HMGA2 reporter plasmid is also shown (upper). (F) Schematic diagram of *HMGA2* promoter region (upper). The regions targeted by the designed primer pairs were shown (amplicon 1 and amplicon 2). TSS, transcription start site. A549 cells stably expressing myc‐VGLL3 were analysed by ChIP assays with anti‐myc or control antibodies to evaluate the binding activity of myc‐VGLL3 to the *HMGA2* promoter region (lower). The percentage input values are shown as relative to that of the control immunoprecipitates. The results represent the mean ± SD (*n* = 3). Asterisks indicate the statistical significance (*p < 0.05) calculated using a Student's *t*‐test

VGLL3 is a cofactor for the TEAD transcription factor family, and TEAD4, a member of this family, is known to be involved in EMT progression.^26^ Therefore, we analysed the role of TEAD4 in VGLL3‐induced EMT‐like phenotypes. TEAD4 knockdown significantly suppressed HMGA2 expression and, in turn, recovered E‐cadherin expression in VGLL3‐expressing cells both at the mRNA and protein levels (Figure 3A,B). TEAD4 knockdown significantly repressed cell motility in these cells (Figure 3C,D). To investigate whether *HMGA2* is a target gene of the VGLL3‐TEAD4 transcriptional complex, we constructed a reporter plasmid, in which enhanced green fluorescent protein (EGFP) expression was driven by *HMGA2* promoter activity. Co‐expression of VGLL3 and TEAD4, but not the expression of a single gene, significantly enhanced this reporter activity (Figure 3E). Chromatin immunoprecipitation (ChIP) assays were also performed to evaluate binding of VGLL3 to the *HMGA2* promoter region. We designed two primer pairs to target the region 2000 bp upstream from the transcriptional start site, and the corresponding region of each amplicon is shown in Figure 3F (amplicon 1, from −2023 and −1821; amplicon 2, from −660 and −484). Owing to the lack of antibodies against endogenous VGLL3, myc‐VGLL3 was precipitated from VGLL3‐expressing A549 cells. ChIP assays (amplicon 2) showed significant association of VGLL3 to the proximal *HMGA2* promoter region (approximately 1000 bp upstream from the transcription start site) (Figure 3F). These results suggest that *HMGA2* is a target gene of the VGLL3‐TEAD4 complex.

### 3.4 VGLL3 contributes to E‐cadherin repression and increases cell motility upon TGF‐β stimulation

**FIGURE 4:**
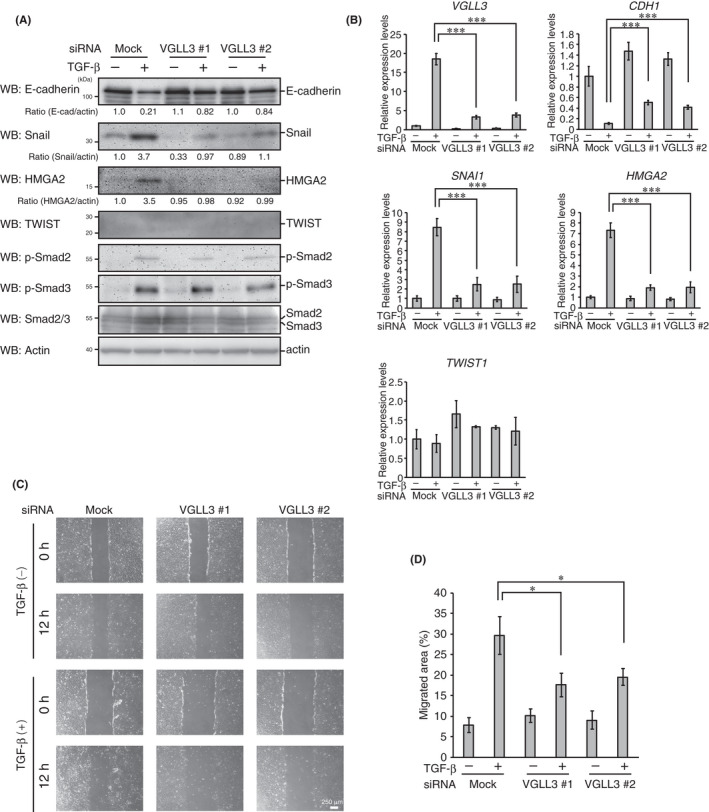
VGLL3 is involved in TGF‐β‐induced EMT‐like phenotypes. (A, B) A549 cells were transfected with the indicated siRNAs for 48 h. After incubation, cells were treated with or without TGF‐β (5 ng/ml) for 48 h. After additional incubation, cells were analysed by Western blotting with the indicated antibodies (A) and analysed for *VGLL3*, *HMGA2*, *CDH1* (E‐cadherin) and *SNAI1* (Snail) mRNA expression using qPCR, as in Figure 2(E) (B). (C) A549 cells were transfected with the indicated siRNAs for 48 h. After incubation, cells were additionally transfected with the indicated siRNAs and stimulated or left untreated with TGF‐β (5 ng/ml) for 48 h. After additional incubation, wound healing assays were performed in the presence or absence of TGF‐β (5 ng/ml). Representative images of wounded areas of A549 cells at 0 and 12 h after wounding are shown. (D) Migrated areas at 12 h after wounding. Error bars show SD (*n* = 3). Asterisk indicates the statistical significance (*p < 0.05) calculated using Student's *t*‐test

Because TGF‐β is a potent EMT‐promoting cytokine known to induce VGLL3 expression, we next examined the role of VGLL3 in TGF‐β‐induced EMT phenotypes. *VGLL3* knockdown significantly repressed E‐cadherin downregulation upon TGF‐β stimulation (Figure 4A,B). Due to the lack of antibodies for endogenous VGLL3 protein, we confirmed siRNA‐mediated *VGLL3* repression by qPCR analysis (Figure 4B). *VGLL3* knockdown also suppressed TGF‐β‐induced upregulation of Snail and HMGA2 without affecting the phosphorylation of Smad2 and Smad3 (Figure 4A,B). Although TWIST was reported to be a downstream target of HMGA2,^27^ TWIST was not induced by TGF‐β stimulation in our experimental condition (Figure 4A,B). Notably, wound healing assays revealed that *VGLL3* knockdown significantly repressed the enhancement of cell motility upon TGF‐β stimulation (Figure 4C,D). These results suggest that VGLL3 contributes to E‐cadherin repression and cell motility enhancement by inducing HMGA2 expression during TGF‐β‐induced EMT progression.

### 3.5 VGLL3 is involved in EMT‐like characteristics in mesenchymal type cancer cells

**FIGURE 5:**
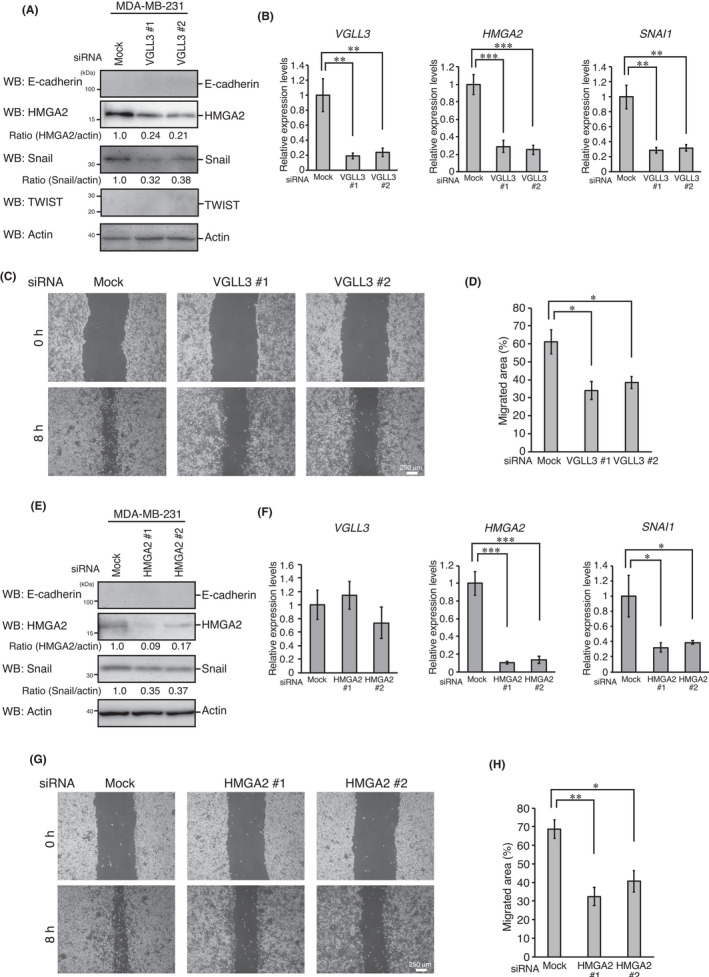
The VGLL3‐HMGA2 axis is involved in cell motility in mesenchymal cancer cells. (A, D) MDA‐MB‐231 cells were transfected with the indicated siRNAs for 48 h. After incubation, cells were analysed by Western blotting with the indicated antibodies. (B, F) MDA‐MB‐231 cells were transfected with the indicated siRNAs for 48 h. After incubation, cells were analysed for *VGLL3*, *HMGA2*, *SNAI1* (Snail) and *TWIST1* (TWIST) mRNA expression using qPCR, as in Figure 2E. (C, G) Representative images of wounded areas of MDA‐MB‐231 cells transfected with the indicated siRNAs at 0 and 8 h after wounding. (D, E) Migrated areas at 8 h after wounding. Error bars show SD (*n* = 3). Asterisk indicates the statistical significance (**p < 0.01, *p < 0.05) calculated using Student's *t*‐test

We previously found that VGLL3 is highly expressed in mesenchymal cancer cells, such as breast cancer MDA‐MB‐231 cells.^15^ Thus, we analysed the role of VGLL3 in EMT‐like mesenchymal characteristics in MDA‐MB‐231 cells. E‐cadherin expression levels were too low to be detected by Western blot analysis (Figure 5A) or qPCR (data not shown) even in *VGLL3* knocked down cells. In contrast, *VGLL3* knockdown significantly repressed Snail and HMGA2 expression at the mRNA and protein levels (Figure 5A,B). TWIST expression was undetectable at the mRNA (data not shown) or protein level (Figure 5A). Notably, *VGLL3* knockdown significantly reduced the motility of MDA‐MB‐231 cells (Figure 5C,D). *HMGA2* knockdown similarly repressed Snail expression and cellular motility, whereas VGLL3 expression was not affected by *HMGA2* knockdown (Figure 5E–H). These results suggest that the VGLL3‐HMGA2 axis is involved in EMT‐like cell motility in mesenchymal cancer cells.

### 3.6 VGLL3 expression is associated with poor prognosis in various types of human cancer patients

**FIGURE 6:**
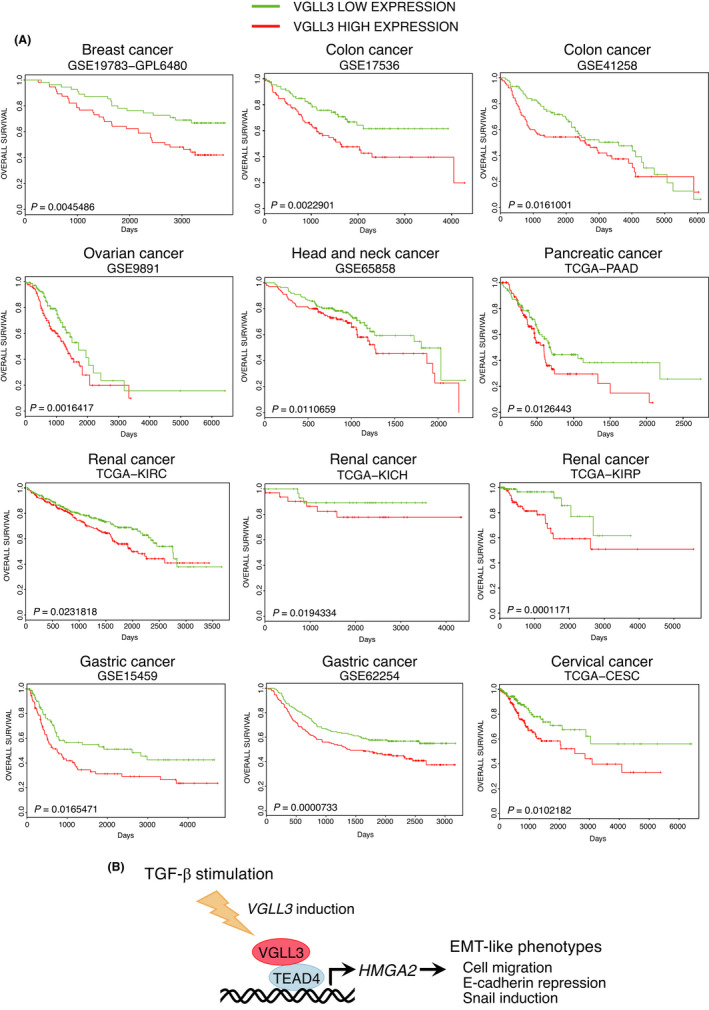
High VGLL3 expression correlates with poor prognosis of various cancer patients. (A) Prognosis data of various cancer patients are shown as Kaplan–Meier plots. Plots were generated by PROGgeneV2 database. Patients were divided into two groups (VGLL3‐high and VGLL3‐low) according to the median value of VGLL3 expression level in each database. Statistical significance was calculated by log‐rank test. The number or name of database is shown above each plot. (B) VGLL3, a cofactor for TEAD4, promotes EMT‐like phenotypic changes, such as enhanced cell motility, E‐cadherin reduction and Snail induction via HMGA2. VGLL3 is involved in TGF‐β‐stimulated cells and mesenchymal cancer cells

EMT is known to contribute to cancer progression.^2^ Therefore, we next examined the association of VGLL3 expression with the prognosis of human cancer patients using the PROGgeneV2 database.^28^ Patients were divided into two groups (VGLL3‐high and VGLL3‐low) according to the median value of VGLL3 expression level in each database. Notably, VGLL3‐high groups showed poor prognosis in databases of various types of cancers such as breast, colon, ovarian, head and neck, pancreatic, renal, gastric and cervical cancer (Figure 6A). We also analysed *VGLL3*, *HMGA2* and *SNAI1* expression in human cancers using the GEPIA database^29^ and found that *VGLL3* expression has a weak but significant positive correlation with *HMGA2* and *SNAI1* expression in esophageal carcinoma, head and neck squamous cell carcinoma and liver hepatocellular carcinoma (Fig. S1). These results suggest that VGLL3‐induced EMT‐like cell motility is associated with cancer progression and poor prognosis in various types of human cancers.

## 4 DISCUSSION

In this study, we found that VGLL3 is involved in EMT‐like characteristics by promoting E‐cadherin repression and cell motility via HMGA2. VGLL3 is also involved in EMT‐like phenotypes in TGF‐β‐stimulated cells and mesenchymal breast cancer cells. VGLL3 was found to be associated with poor prognosis in various types of cancer patients. These results suggest that the VGLL3‐HMGA2 axis is associated with cancer progression by increasing cell motility in cancer cells (Figure 6B).

E‐cadherin is a component of the adherence junction, and loss of E‐cadherin expression lowers cell adhesion and increases cell motility.^30^ Because VGLL3 expression affected both E‐cadherin expression and cell motility, VGLL3 seems to stimulate cell motility via E‐cadherin repression. However, recovery of E‐cadherin expression through *VGLL3* knockdown was not apparent in MDA‐MB‐231 cells, even though cell motility was significantly repressed. Therefore, we speculate that E‐cadherin repression is not the only mechanism that regulates cell migration in VGLL3‐induced increase in cell motility.

We found that *HMGA2* is a target gene of the VGLL3‐TEAD4 complex and is involved in VGLL3‐dependent cell migration. HMGA2 is a DNA‐binding protein that regulates gene expression.^31^ Consistent with previous results, *HMGA2* knockdown reduced Snail expression, indicating that HMGA2 is an upstream regulator of Snail.^24^ However, Snail knockdown did not affect EMT‐like phenotypes in VGLL3‐expressing cells, suggesting that Snail is not a major EMT inducer under these conditions. Recent studies have shown that IGF2BP2‐Ras‐ERK signalling plays a pivotal role in cancer cell metastasis and acts downstream of HMGA2.^32^ Given that ERK activity is upregulated in VGLL3‐expressing cells,^15^ we hypothesized that IGF2BP2‐Ras‐ERK signalling is involved in the upregulation of cell motility and acts downstream of the VGLL3‐HMGA2 axis. Previous studies have shown that HMGA2 is a direct target of Smad proteins in TGF‐β signalling.^24^, ^25^, ^27^ Given that VGLL3 binds to the *HMGA2* promoter region and that VGLL3 knockdown repressed TGF‐β‐induced HMGA2 expression, Smad proteins and VGLL3 are likely to act synergistically on the *HMGA2* promoter and effectively induce *HMGA2* expression.

In general, EMT is associated with the induction of the mesenchymal marker N‐cadherin as well as the reduction of the epithelial marker E‐cadherin, together with the induction of cell motility.^33^ However, VGLL3‐expressing cells showed only E‐cadherin repression, and no apparent change was observed in N‐cadherin expression, even though cell motility increased. Based on these observations, we hypothesized that VGLL3 is an inducer of partial EMT. Emerging evidence has revealed that partial EMT, rather than complete EMT, plays a vital role in cancer progression.^34^, ^35^ Consistent with this hypothesis, our data showed that VGLL3 expression was positively correlated with poor prognosis in various types of cancer patients. We speculate that VGLL3 functions as an inducer of partial EMT in cancer cells by stimulating cell motility and accelerating cancer progression.

VGLL3 is highly expressed in mesenchymal cancer cells, such as MDA‐MB‐231 cells.^15^ However, the regulation of its expression in these cells remains obscure. Because VGLL3 is a downstream target of TGF‐β‐Smad3 signalling,^13^ aberrant activation of this signalling may be a cause of the high expression of VGLL3. Cancer cells have dysregulated activation of various signalling pathways related to EMT progression, such as the Wnt pathway,^36^ the NF‐κB pathway^37^ and the Jak/Stat pathway.^38^ Thus, it is possible that these signalling pathways also regulate VGLL3 expression. Furthermore, because VGLL3 expression is affected by epigenetic status,^13^ epigenetic changes in cancer cells may be involved in its expression.

In conclusion, we found that VGLL3 promotes E‐cadherin repression and cell motility via HMGA2 in TGF‐β‐stimulated and mesenchymal cancer cells. Furthermore, we found that VGLL3 expression correlated with poor prognosis in various types of cancer patients. To develop VGLL3 inhibitors for cancer treatment, further analyses regarding the molecular mechanisms underlying VGLL3 activation and VGLL3‐dependent cell motility are required.

## CONFLICT OF INTEREST

The authors declare that they have no conflicts of interest regarding the contents of this article.

## AUTHOR CONTRIBUTIONS

**Naoto Hori:** Investigation (equal); Writing – original draft (equal). **Yuki Takakura:** Investigation (equal); Writing – original draft (equal). **Ayumi Sugino:** Investigation (equal). **Shuto Iwasawa:** Investigation (equal). **Kota Nomizo:** Investigation (equal). **Naoto Yamaguchi:** Data curation (equal); Resources (equal). **Hiroyuki Takano:** Data curation (equal); Resources (equal). **Noritaka Yamaguchi:** Conceptualization (lead); Investigation (supporting); Methodology (lead); Supervision (lead); Writing – review & editing (lead).

## Supporting information

Figure S1

## Data Availability

All data used to support findings of the study are available from the corresponding author upon reasonable request.
