# Supplementary material for: Vestigial‐like family member 3 stimulates cell motility by inducing high‐mobility group AT‐hook 2 expression in cancer cells
Source: J Cell Mol Med. 2022 Apr 2;26(9):2686–97. doi: 10.1111/jcmm.17279 (PMC9077286; doi:10.1111/jcmm.17279)
Supplement: Supplementary file 1 — Figure S1 [file JCMM-26-2686-s001.pdf]

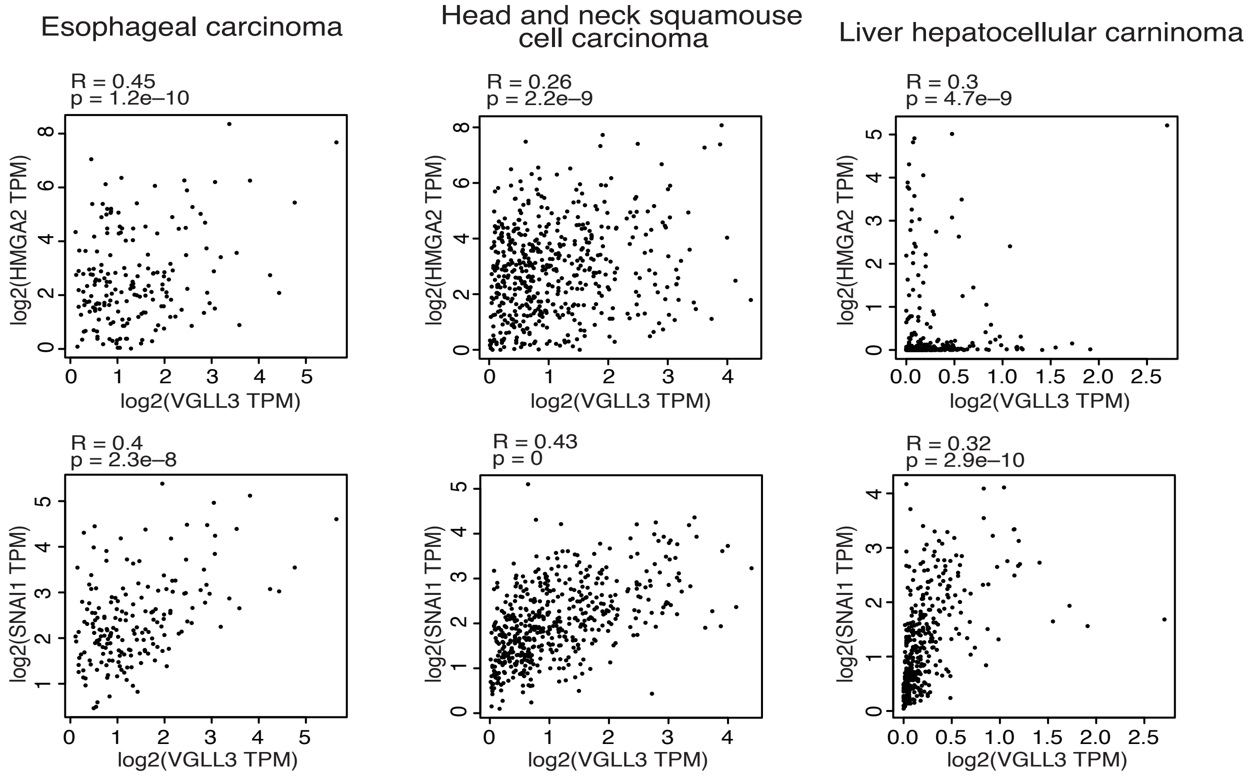

Supplemental Fig. S1

Expression levels of *VGLL3*, *HMGA2*, and *SNAI1* were analyzed by the GEPIA database. Positive correlaitons between *VGLL3* and *HMGA2* and between *VGLL3* and *SNAI1* were detected in esophagieal carcinoma, head and neck squamouse cell carcinola, and liver hepatocellular carcinoma.

Supplemental Fig. S1
